# Creating an ethics curriculum using a structured framework

**DOI:** 10.5116/ijme.58ed.e607

**Published:** 2017-04-25

**Authors:** Lawrence Cheung

**Affiliations:** 1 Department of Medicine, University of Alberta, Canada

**Keywords:** Ethics curriculum, structured framework, Roff’s structured learning in clinical ethics, SLICE, Canada

## Introduction

While patients and their families often expect their physicians to discuss the options in end-of-life care,^1^ medical students and residents often feel unprepared to perform this task in clinical practice.^2^ At times, health care providers may disagree with patients or their families about the direction this end of life care should take. Learners often desire more organized instruction during their medical training to teach them how to elicit their patients’ views and engage patients and families in these end-of-life discussions.^3^ Thus, it is important to incorporate teaching in this and other medical ethics topics into the medical education curriculum.

However, medical educators may find it difficult to develop and implement a curriculum in medical ethics if they lack a structured framework to guide them.^4^ Roff’s structured learning in clinical ethics, also known as the SLICE model,^5^ is an example of a structured framework that educators can use to create a medical ethics curriculum.

The purpose of this article is to show medical educators how they can use the SLICE framework to develop their own medical ethics curriculum. To demonstrate this, we will illustrate how our respiratory residency program has used this framework to structure our curriculum in the end of life communication and care, which is one of the subjects in our overall medical ethics curriculum.

### Issues Faced While Developing the Curriculum

The SLICE framework divides learning content into five areas or domains. These domains include compliance, concurrence, conversation, conversion, and conscience.^5^^, ^^6^ When educators are constructing a medical ethics curriculum, each topic in the curriculum should include content in these five domains to ensure that all aspects of the topic are comprehensively covered. This article will now describe each of these curricular domains in further detail, using the topic of end of life communication and care as an example. 

The compliance domain refers to the regulations, laws, or social mores surrounding the ethical topic. For the end of life communication and care, we first covered the issues around decision making. We taught how to assess a patient’s capacity to make informed decisions, what to do when a patient lacks capacity, and how to reconcile disagreements between the health care team and the patient’s surrogate decision makers over the direction of the end of life care. We used reading assignments and case scenarios to discuss this legislation as it applies to a patient’s right to refuse life-sustaining treatment, withdrawal of active treatment, palliation at home or a hospice facility, and continuation of life-sustaining treatments contrary to the recommendations from the health care team. Then, we covered the steps involved when a patient wishes to construct a personal directive and advanced care plan or wants to designate a substitute decision maker. 

The concurrence domain reviews how to provide care to patients who may have different points of view including different cultural, religious, and social perspectives.  Using case scenarios, reading assignments, and self-reflective essays, our curriculum covered different religious and cultural perspectives regarding the style of the end of life communication and preferences for care. For example, we examined different beliefs regarding truth-telling and breaking bad news, the role of families in end of life care, and the components of a “good death”.

At the same time, we were careful to avoid the negative consequences of “othering”,^7^ where emphasizing the differences of other groups fosters a tone of superiority rather than understanding. Our curriculum also explored the meaning of “cultural competence”^8^ and its role at the end of life care. To avoid simply reinforcing stereotypes, we stressed the importance of good communication to ascertain their patients’ treatment preferences, regardless of their patients’ religious or cultural affiliation.

The conversation domain covers how to discuss ethical and moral issues with patients while respecting their viewpoints. As Covey once noted, “Most people do not listen with the intent to understand; they listen with the intent to reply”.^9^ Thus, our curriculum reviewed how to actively listen to patients, effectively communicate with patients of different cultures, and show empathy. To deliver this content, we used small group seminars and role play.

The conversion domain reviews how to settle on a clinically beneficial management plan with the patient while respecting the patient’s own views. In our curriculum, we covered how to effectively negotiate with patients or their surrogate decision makers, manage conflict within the health care team, and establish trust with the patient. We emphasized the importance of finding common ground between disparate views and establishing goals with which everyone – patient and health care team - can agree. And, we reviewed when it may be necessary to initiate a dispute resolution process or provide time-limited interventions to further these goals. To deliver this content, we used reflective essays, written assignments, and small group discussion.

In the conscience domain, learners take their clinical workplace experiences and add them to the lessons learned in the compliance, concurrence, conversation, and conversion domains of the curriculum.  Thus, the curriculum should allow the learners to reflect on the material discussed and presented to them. Our curriculum used tools such as reflective essays and small group discussions to help encourage reflective thought. We also emphasized that the process will continue to evolve throughout their careers and that uncertainty is often a part of clinical and ethical practice.

### Lessons Learned While Implementing the Curriculum

One year after implementing our curriculum in the end of life communication and care, we evaluated our experience and sought feedback from the residents. They praised the structured teaching. It enabled them to gradually construct a cognitive scaffold for their knowledge as the lessons progressed from one domain to the next in the SLICE framework. What had previously been passive learning in seminars transformed into active learning in the small group discussions which both faculty and residents enjoyed? The residents felt more at ease in conducting end life communication in actual clinical settings, and they, in turn, were confident in role modeling this behavior to junior residents and medical students. Also, by explicitly teaching end of life communication in a deliberate fashion, the residents sensed that we valued this skill on par with traditional medical knowledge and procedural skills. In other words, we legitimized the topic as necessary for their clinical practice. They were eager for us to implement this structure to other topics in our ethics curriculum.

As an unexpected benefit, faculty who led the group discussions gained a new perspective on their views as the residents often challenged them to justify their opinions.  The faculty also built on their knowledge and dispelled some of their misconceptions about conducting end of life communication. Consequently, they performed this skill with more comfort and acted as role models to the rest of the health care team.

## Conclusions

The SLICE framework is a useful tool to structure curricular content to teach end of life communication and care. International medical educators can use this framework to construct curricula for other areas within medical ethics^5^ or in other specialties in medicine.^6^ The framework does not propose a single “correct” way to conduct ethical behaviour because proper ethical conduct differs throughout the world based on local laws, customs, and attitudes. Rather, the framework gives medical educators in different parts of the world a sequence and structure they can follow to teach appropriate ethical behaviour specific to their region.

Based on feedback from our residents, their knowledge and skills in end of life communication and care has improved. We plan on using other rubrics^10^ to assess whether there has been any change in their reflection and attitudes as well. We have already started to implement the SLICE framework to teach other topics in ethics. To determine if the residents’ self-perceived improvement in behaviour has affected the actual clinical outcome, future research will survey families and patients about their satisfaction with the residents’ ethical conduct.

### Conflict of Interest

The author reports no conflicts of interest in this work.

## References

[1] Walczak A, Butow PN, Davidson PM, Bellemore FA, Tattersall MHN, Clayton JM, Young J, Mazer B, Ladwig S, Epstein RM (2013). Patient perspectives regarding communication about prognosis and end-of-life issues: How can it be optimised?. Patient Education and Counseling.

[2] Buss MK, Marx ES, Sulmasy DP (1998). The preparedness of students to discuss end-of-life issues with patients.. Acad Med.

[3] Ury WA, Berkman CS, Weber CM, Pignotti MG, Leipzig RM (2003). Assessing medical students' training in end-of-life communication: a survey of interns at one urban teaching hospital.. Acad Med.

[4] Lang CW, Smith PJ, Ross LF (2009). Ethics and professionalism in the pediatric curriculum: a survey of pediatric program directors.. Pediatrics.

[5] Roff S, Norman J. Structured Learning in Clinical Ethics (SLICE) for teaching informed consent for paediatric anaesthesia. Bulletin of the Royal College of Anaesthetists. 2010;62:36-38.

[6] Webster S. How to teach reflective ethical practice in postgraduate gastroenterology: the SLICE framework. Frontline Gastroenterology. 2013;4(2):143-146. 10.1136/flgastro-2012-100249 PMC5369809 28839716

[7] Johnson JL, Bottorff JL, Browne AJ, Grewal S, Hilton BA, Clarke H (2004). Othering and being othered in the context of health care services.. Health Communication.

[8] Paul D, Ewen SC, Jones R (2014). Cultural competence in medical education: aligning the formal, informal and hidden curricula.. Adv Health Sci Educ Theory Pract.

[9] Covey SR. Habit 5: seek first to understand, then to be understood. The 7 habits of highly effective people: powerful lessons in personal change. 2nd ed. New York, NY: Simon & Schuster; 2004.

[10] Wald HS, Borkan JM, Taylor JS, Anthony D, Reis SP (2012). Fostering and evaluating reflective capacity in medical education: developing the REFLECT rubric for assessing reflective writing.. Acad Med.

